# Impact of High Mathematics Education on the Number Sense

**DOI:** 10.1371/journal.pone.0033832

**Published:** 2012-04-25

**Authors:** Julie Castronovo, Silke M. Göbel

**Affiliations:** 1 Department of Psychology, University of Hull, Hull, United Kingdom; 2 Department of Psychology, University of York, York, United Kingdom; Cuban Neuroscience Center, Cuba

## Abstract

In adult number processing two mechanisms are commonly used: approximate estimation of quantity and exact calculation. While the former relies on the approximate number sense (ANS) which we share with animals and preverbal infants, the latter has been proposed to rely on an exact number system (ENS) which develops later in life following the acquisition of symbolic number knowledge. The current study investigated the influence of high level math education on the ANS and the ENS. Our results showed that the precision of non-symbolic quantity representation was not significantly altered by high level math education. However, performance in a symbolic number comparison task as well as the ability to map accurately between symbolic and non-symbolic quantities was significantly better the higher mathematics achievement. Our findings suggest that high level math education in adults shows little influence on their ANS, but it seems to be associated with a better anchored ENS and better mapping abilities between ENS and ANS.

## Introduction

Number processing is part of a wide range of numerical activities such as counting, estimating, simple addition as well as more complex mathematical activities such as solving equations. It is often assumed that higher mathematical abilities, typically acquired years later than basic number processing and calculation, rest upon proficiency in early numerical activities (e.g. counting, reciting multiplication tables). Consequently it is often expected that expertise in mathematics goes hand in hand with excellent basic number processing abilities. In a restaurant, for example, it falls typically onto the mathematics lecturer to sort out the bill for a larger group. However, it is not clear yet whether mathematics expertise and excellent basic number processing and calculation skills are as closely linked as commonly assumed. The current paper investigates whether undergoing an education dedicated to mathematics at a high level is related to a better general grasp of numbers and to excellent basic numerical abilities.

### The Approximate Number System

Over the last 30 years, research in numerical cognition has gathered strong evidence that humans are equipped with a core ability to grasp numerical quantities, commonly referred to as the “number sense” [1] or the approximate number system (ANS) [2]. This core ability seems to be part of an ontogenetic and phylogenetic early system, as it has already been found in newborns [3] and in various animal species [4]. The number sense has been largely associated with a specialized cerebral system located in the intraparietal sulcus (IPS) of both hemispheres (see [5] for a review). It is defined as intuitive, as it is fast, automatic and inaccessible to introspection [6]. It is also approximate because it obeys Weber’s law at a behavioral and at a neuronal level [7]: the larger the numbers/numerosities, the more approximate their processing.

The metaphor of a logarithmically compressed mental number line has been commonly used in the literature to refer to number representations and can be thought of a conceptualization of how numerical processing obeys Weber’s law. According to this metaphor, numerosities are represented in an analogous format by overlapping Gaussian distributions of activation [1]. Weber’s law is recurrent in numerical cognition, as it has been repeatedly found: 1) in different populations, such as in adults in Western (e.g., [8]–[9]) or remote cultures (e.g., [10]–[11]), in pre-verbal infants (e.g., [12]–[13]) and children (e.g., [14]–[15]), as well as in different animal species, such as rats (e.g., [16]) or rhesus monkeys (e.g., [17]); 2) in different tasks: comparison (e.g., [7]); estimation (e.g., [18]); arithmetical operations (e.g., [19]–[20]); and 3) for symbolic and non-symbolic numerical material [21]. Moreover, the pervasiveness of Weber’s law is also shown at the neural level in children and adults, as well as across species: brain responses are similarly tuned to approximate numerosity in human adults [7], 3-month-old infants [22], and in macaque monkeys [23].

### The Weber Fraction

**Figure 1:**
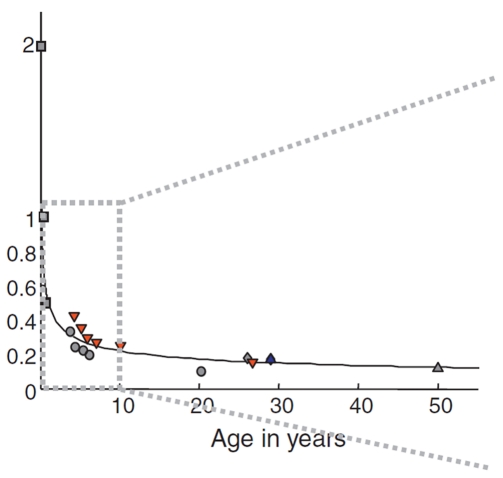
ANS acuity over the lifespan. ANS acuity is measured by estimated Weber fractions as a function of age. The solid black line represents the power function fit. Reprinted from Piazza (2010) [28].

Recently, the Weber fraction (*w*) has been used to assess the ANS acuity. This measure constitutes a sensitive and relevant quantitative estimator of the amount of error of the ANS, as it corresponds to the standard deviation of its estimated Gaussian distribution [7],[11],[24]–[25]. Research on the development of the Weber fraction is only in its outset, but several recent studies suggest that, in typical development, the Weber fraction decreases with age: it follows a trajectory with an initial sharp decrease in infancy (corresponding to an increase in acuity), followed by a gradually smaller but ongoing reduction over time (see [5] for a review) (see Figure 1). The average *w* for educated numerate adults is estimated to be between 0.11 and 0.15 [2], [6], [26]. The cause of the decrease of *w* with age, corresponding to an increase in ANS precision, is still unknown and discussed in the literature [26]. A first hypothesis is that it could be the result of a simple maturational process, as no cultural or educational factors can account for the developmental trend of *w* found in preverbal infants. A second hypothesis is that experience individuals have with numerical information, for example through the acquisition of the symbolic number system and a day-to-day engagement in numerical discrimination, would explain the greater ANS precision. Pica et al.’s [11] results indicate a slightly larger *w* in a remote culture (the Munduruku: *w* = 0.17), than in an educated numerate Western culture (French: *w* = 0.12), supporting this latter hypothesis ([8]; but see [5], [26]). Moreover, the impact of experience on the ANS has recently been illustrated in blind people: early visual deprivation and its following experience with numerical information seem to have a positive impact on numerical estimation abilities [27]. Therefore, it is reasonable to assume that these two hypotheses are not exclusive, and that maturation processes, as well as education and experience with numerical information, could be responsible for the refined precision of *w* with age [3].

### The ANS and Mathematics Achievement

Different proposals have been made for the acquisition of symbolic numerical knowledge (see Piazza [28] for a review). Counting and the acquisition of its principles have been highlighted as a critical foundation for later math achievement, in particular arithmetic that develops in the pre-school years [29], [30]. Mastery of the how-to-count principles has notably been found to predict children’s later abilities in math and in particular in arithmetic [31]. The ANS has also been considered as essential for the acquisition of symbolic numerical knowledge (e.g., [32], [33]), while other authors focus on the object-tracking system (OTS), as a core quantitative system allowing the exact representation of small numerosities with an upper limit of 3–4 objects [34], [35]. A combination of the ANS and the OTS has also been proposed [36], [37]. However, it has also been postulated that neither the ANS, nor the OTS play a significant role in acquiring symbolic numerical knowledge [38], whereas Butterworth [39] proposed the existence of an innate and exact representation for large numbers.

Nevertheless, the idea that the ANS is crucial for the acquisition of subsequent numerical and arithmetical skills (e.g., [1]–[2], [19]) seems to have gathered greater evidence, especially in view of recent research findings (see [28], for a review). Most of the research has been conducted in children, studying the relationship between mathematics achievement and tasks involving basic numerical abilities. A growing set of data has shown that mathematics achievement and/or counting abilities in children correlate with estimation abilities [24], [40]–[46], non-symbolic arithmetic performances [19], symbolic and non-symbolic comparison accuracy [43] and the symbolic numerical distance effect (i.e., the greater mathematics achievement, the smaller the symbolic distance effect) [43], [47], [48]. However, several studies have failed to find a significant association between the ability to deal with approximate numerosities and arithmetic [49], [50]. In contrast to other proposed precursors of arithmetic such as counting, the mechanisms by which ANS acuity could form the basis of arithmetic are currently underspecified. Butterworth [51], for example, highlights that it is unclear how an increase in ANS precision could help to establish one-to-one correspondence, an important step in learning to count, add and subtract. Thus, although the ANS might provide a primary foundation for mathematical skills, many researchers argue that this system alone is not sufficient for the development of arithmetic.

However, it is important to establish whether ANS acuity is related to higher order mathematics. Only a few studies have directly investigated the link between the ANS acuity (*w*) and higher order mathematics and numerical abilities so far. First, Halberda et al. [2] showed that individual differences in 14-year-old teenagers’ *w* correlate with their past scores on mathematics tests all the way back to kindergarten. Second, Piazza et al. [26] provided evidence of the foundational role of the core ANS for the development of higher level numerical abilities, by showing the existence of a link between dyscalculia and an impaired ANS. Moreover, the severity of dyscalculic children’s ANS impairment appeared to be a good predictor of their defective performances on symbolic comparison tasks. However, and contrary to the authors’ predictions, *w* did not correlate with the children’s mathematics achievement. Supporting these latest results, Mazzocco, Feigenson & Halberda [52], conducted a study on ninth grade students with either dyscalculia or with low, typical or high mathematics achievement and also found that dyscalculia is linked with an impaired ANS acuity. In addition, their results showed that dyscalculia is associated with impaired mapping abilities between ANS and number words. To estimate the precision of the mapping between the ANS and the symbolic verbal number system, Mazzoco et al. [52] submitted their participants to a number identification task, in which participants had to verbally estimate the numerosity of sets of dots briefly presented. This task involves a non-symbolic to symbolic mapping. Moreover, in this study the level of achievement in 14–15 year-olds without dyscalculia did not correlate with ANS acuity per se (*w*), but it did with the precision of verbal mapping. Finally, Mazzocco, Feigenson & Halberda [53] demonstrated that mathematics ability in primary school can be predicted by the ANS acuity measured at preschool, prior to formal education in mathematics.

These findings speak for the idea that ANS might play an important role in the normal development of more advanced numerical and mathematical abilities. However, the reverse seems also plausible given that the acquisition of the symbolic number system is in turn assumed to result in deep changes in our primary core quantity system, the ANS [33].

### The Exact Number System

Recent studies strongly suggest that the acquisition of more advanced numerical skills with development might shape the ANS [6]. Indeed, with the acquisition of the symbolic number system, children develop mapping routes between the numerical symbolic codes and their corresponding core non-symbolic magnitude representations. The greater their counting and mathematical abilities, the greater are their mapping abilities [13], [24], [43], [47], [54]. However, the acquisition of the symbolic number system does not simply induce the development of mapping routes between the symbolic number codes and the ANS, it also involves profound changes in the cerebral network responsible for numerical processing: a progressive shift from predominance of the right IPS to involvement of both left and right IPS for symbolic and non-symbolic numerical processing [21]–[22], [55], [56] and an increasing activation in and around the left IPS for arithmetic processing [57] have been reported with increasing age. These changes have recently been interpreted as reflecting a progressive refinement of the ANS into a second number system dealing with symbolic numbers for exact number processing, named here the exact number system (ENS).

A large set of behavioural data support the hypothesis of a refinement of the ANS into the ENS over development with the acquisition of symbolic numerical knowledge. The existence of the ENS as a semantic numerical representation with greater precision to process symbolic numerals has firstly been suggested with the observation that the distance and size effects for symbolic numerals are smaller than for non-symbolic numerosities [58]. More recently, children showed different patterns of performance over development, when performing number line tasks. Indeed, in kindergarten, children presented approximate performances, reflecting the use of a logarithmic numerical representation (i.e., the ANS) to solve number line tasks. Second and third graders presented different patterns of performances according to the numerical range tested: precise performances on 0–100 number lines, approximate performances on 0–1000 number lines. These findings suggest the existence of multiple semantic numerical representations, such as the ANS and the ENS. Moreover, they reflect the simultaneous co-existence at a certain point in development (i.e., from second grade) of an immature early developing approximate logarithmic numerical representation (i.e., the ANS) for unfamiliar larger numerical range and a more mature late-developing precise linear numerical representation (i.e., the ENS) for familiar smaller numerical range. On the other hand, adults presented linear numerical processing in both numerical ranges: 0–100 and 0–1000 number lines [46], [59]–[62]. These results have been recently replicated by Ashcraft & Moore [63], by submitting first to fifth graders and college students to number line estimation tasks. Their results showed an increasing occurrence of a linear pattern of performance with age. Moreover, Ashcraft & Moore [63] found that children’s mathematics achievement significantly correlated with the strength of linear responding in number line tasks. According to these authors, education and the acquisition of symbolic number knowledge play a crucial role in the emergence of the ENS from the ANS (e.g., [46], [63]).

Further behavioural evidence on the development of ENS with age comes from the size congruity effect found in number stroop paradigms [64]. In this paradigm, a pair of Arabic digits is presented to the participants, who have to either respond to the numerically or physically larger number, while ignoring the irrelevant dimension to solve the task. This stroop paradigm can be used to investigate the automatic access to the semantic numerical information from the presentation of symbolic numerals (i.e., the automatic access to the ENS). The automatic activation of the ENS has been found to develop gradually in children, as no size congruity effect could be found in first graders, while it started to be present in third graders and was significantly robust in fifth graders [65], [66].

Neuroimaging data also support the idea of the existence of the ENS in addition of the ANS. Following the observation that, in adults, the estimated ANS acuity at the neuronal level is greater in the left IPS (*w* = 18) than in the right IPS (*w* = .25) [7], it has notably been suggested that the ENS might be lateralized in the left hemisphere [5]–[6], [21], [49], [67], [68]. Moreover, it appeared that Arabic numerals are coded with greater precision than sets of objects, particularly in the left hemisphere [21]. With the use of an fMRI adaptation paradigm, Cohen Kadosh et al. [69] found evidence for format dependence, as the neuronal modulatory effect of magnitude change appeared to vary with the format of presentation (symbolic: Arabic numerals vs. non-symbolic: sets of dots). These results support the existence of different semantic numerical representations in adults’ IPS for symbolic numerals (i.e., the ENS) and non-symbolic numerosities (i.e., the ANS).

Therefore, with the acquisition of arbitrary cultural symbols for numbers, our core ANS would be refined in the left parietal lobe into a formal, symbolic and linearly represented ENS [6], [33], allowing the automatic access from symbolic numbers to their corresponding magnitude; while a dormant approximate logarithmic number system would remain in the right IPS [6], [52], [59]. Nowadays, little is known about the effects of mathematical education on the ENS. The aim of our study was to test in adults the effects of high level of mathematics education on the two core systems of number knowledge, the ANS and the ENS, as well as on the mapping abilities between them.

### The Current Study

To date, the study of our core number systems, the ANS and the ENS, has just begun and many questions remain open. Evidence of the link between the ANS and higher-level mathematics abilities is still sparse and inconclusive, especially in adults. The next challenge, as recently highlighted by Dehaene [6], is to understand how math education changes our two core systems of number knowledge (ANS and ENS), and to investigate whether a sustained education in mathematics involves greater ANS acuity [8]. Here, we directly addressed the question of the impact of high level education in mathematics on the ANS, the ENS and the mapping abilities between these two core number systems, by comparing performance on basic numerical tasks with symbolic and non-symbolic material, in a group of participants who are studying mathematics at university, and can thus be considered as a group with an extended instruction in mathematics, to a control group. Both groups of participants were initially submitted to a series of arithmetic tests to measure their level of mathematics achievement, as well as to a spelling test as a control measure for more general cognitive abilities (see for example [19] for a similar use of verbal tasks as control measures). Secondly, participants were respectively tested on: a) a basic non-symbolic numerical comparison task based on Piazza et al. [7], allowing participants’ ANS acuity (*w*) to be measured; b) a symbolic numerical comparison task, allowing to investigate the impact of high level education in mathematics on the ENS; c) a numerosity production and a numerosity perception tasks based on Crollen et al. [18], involving the estimation of non-symbolic quantities either through production (i.e., mapping from the ENS to the ANS) or through perception (i.e., mapping from the ANS to the ENS), allowing the study of the impact of an extended education in mathematics on mapping abilities between the symbolic numerical representations and their corresponding magnitudes.

Recent findings on children with dyscalculia [26], [52] and older children [2] provide strong evidence that the ANS plays an important role in the acquisition of more advanced numerical and mathematical abilities. However, the question of the link between high level mathematics education and ANS acuity is still wide open, especially in adults. If the level of education in mathematics is linked with ANS acuity, then greater ANS acuity (i.e., smaller *w* in the non-symbolic comparison task, smaller coefficients of variation on the numerosity production and perception tasks) should correlate with higher mathematics achievement which in turn is often associated with an extended education in mathematics. Links between mathematics achievement and basic numerical skills on symbolic material, as well as between mathematics achievement and mapping abilities between the ANS and the ENS have been clearly demonstrated in developmental studies [24], [26], [43], [47], [48], [52]: the greater mathematics achievement in children, the greater their symbolic numerical and their mapping abilities between the ANS and the ENS. Can these results be extended to adults? This question is particularly important, as following the observation that the relationship between mathematics achievement and the symbolic distance effect declines with age, it has been postulated that basic number knowledge might only be predictive of mathematics performances at an early age (i.e., when formal mathematics instruction is introduced) [48]. This would be consistent with a hypothesis emphasizing education rather than a purely developmental hypothesis. If the results found in children on the relationship between mathematics achievement and symbolic numerical and mapping abilities can be extended to adults, then participants with a high level of mathematics education should possess a greater ability to compare two numerals (faster reaction times, greater accuracy and/or smaller symbolic distance effect), as well as greater mapping abilities between symbolic numerals and their corresponding magnitudes, i.e they should show a better performance on tasks that rely on the ENS.

No clear predictions, however, can be made yet on whether the effect of a greater experience with the symbolic number system and mathematics would be restricted to better symbolic numerical processing, or whether positive correlations would generalize to non-symbolic numerical processing (as a marker of the ANS) as well. Indeed, regarding this particular issue, different patterns of performances have been found in the literature. On the one hand, some authors found a link between non-symbolic numerical processing (e.g., non-symbolic arithmetical abilities) and mathematics achievement in children [2], [19], [26]. On the other hand, other authors found a particular relationship between numerical processing and mathematics achievement, which was limited to symbolic numerical input and was not found for non-symbolic numerical input [43], [48], [49], [50]. No clear predictions can notably be made on mathematics achievement and the non-symbolic distance effects, as it appeared in children that mathematics achievement was essentially correlated with the symbolic distance effect, while no correlation has been found between mathematics achievement and the non-symbolic distance effect [43], [48]. Therefore, in our study, if an extended experience with mathematics has a general positive impact on all numerical abilities whether they rely on the ENS, the ANS or both, then mathematics achievement should be positively correlated with performances in all our tasks, whatever their format of presentation. On the contrary, if an extended experience with mathematics has a limited positive impact on the ENS and/or its access, then mathematics achievement should only be positively correlated with performances in the tasks involving the ENS, but not on non-symbolic numerical performances per se, neither on the ANS’ acuity.

To sum up, our goal was to determine, in adults, the effects of math education on the two core systems of number knowledge, the ANS and the ENS. In particular, we wanted to investigate whether math education enhances ANS acuity and leads to better basic numerical abilities on symbolic and non-symbolic material, as well as to better mapping abilities between symbolic numerals and their corresponding magnitude representations.

The present study consisted of a series of standardized and empirical tests. The arithmetic subtest of the fourth Wide Range Achievement Test (WRAT4) [70] and separated speeded calculation exercises (i.e., additions, subtractions and multiplications) were used to assess participants’ mathematics achievement. The WRAT4 spelling subtest was also given [70]. These tests were followed by four computerized basic numerical tasks on symbolic and non-symbolic material. The first experiment was a replication of Piazza et al.’s [7] non-symbolic comparison task (Experiment 1), allowing participants’ Weber fraction (*w*) to be computed. The second experiment was a classic symbolic comparison task to a fixed standard (65), allowing participants’ symbolic distance effect to be measured (e.g., [71]) (Experiment 2). The last two experiments then involved the estimation of non-symbolic quantities either through production (Experiment 3) or perception (Experiment 4), so that participants’ mapping abilities in both directions (i.e., from symbolic to non-symbolic; from non-symbolic to symbolic) could be examined. The same participants took part in the different tests and experiments in two experimental sessions, except for the symbolic comparison task (Experiment 2) which included new participants, as this experiment was introduced at a later stage in the study.

### Tests on Mathematics Achievement and Spelling Abilities

#### Participants

The participants in the math group were 34 students (19 males, 15 females; 31 right-handed, 3 left-handed) in the School of Mathematics at the University of Leeds, aged between 19 and 37 years (*M* = 22, *SD* = 4). All participants were either undergraduate students in their final year or post-graduate students (23 undergraduates, 11 postgraduates). The control participants were 37 students (4 males, 33 females; 32 right-handed, 5 left-handed) in the Institute of Psychological Sciences at the University of Leeds aged between 19 and 26 years (*M* = 21, *SD* = 2).

All participants received an information sheet on the study and provided written and informed consent before undertaking the different experiments. All procedures were approved by the ethic committee of the University of Leeds. Participants were offered £9 per session in exchange for their time.

### Tests and Procedure

Mathematics achievement was measured using the standardized math computation subtest of the WRAT4 [70] and a non-standardized investigator-designed calculation test, based on the Graded Difficulty Arithmetic Test (GDA) [72]. These two types of mathematical tests allowed us to compute a global mathematics achievement index per participant. Participants’ spelling ability was also assessed using the standardized spelling subtest of the WRAT4 [70]. This task was introduced as a control task, reflecting more general cognitive processes.

The WRAT4 math computation subtest (green form) consisted of 40 math exercises including additions, subtractions, multiplications and divisions to be answered in written format. The problems are easy at the beginning and then get harder. Participants have to solve as many problems as they can in 15 minutes. The number of exercises solved correctly is reported as raw score.

In the calculation test, subjects had to answer in written format arithmetic problems presented as Arabic digits. This test presented three sections of exercises: 1) additions; 2) subtractions; 3) multiplications. Each section consisted of fifty questions, including two examples, and was divided into two sub-sections. The first sub-sections were made of simple arithmetical exercises, such as “4+6 = ” for the addition section, “4–3 = ” for the subtraction section and “3×3 = ” for the multiplication section. The second sub-sections presented more complicated exercises, such as “22+49 = ”, “64–16 = ” and “12×8 = ” for the addition, subtraction and multiplication sections respectively. Each sub-section had a particular time limit (addition/subtraction sub-sections: 15 sec. and 45 sec.; multiplication sub-sections: 20 sec. and 2 min.), under which participants had to complete as many questions as possible.

The WRAT4 Spelling test (part two) consisted of 42 words for the participants to spell in written format. Each word was first read in isolation, it was then read in a sentence to illustrate its correct use, and read again. Words could be repeated if necessary. The raw score for spelling is the number of words spelled correctly.

### Results

A mathematics achievement index was computed for each participant by averaging scores of the WRAT4 math computation subtest with scores of the three calculation sections (i.e. addition, subtraction, multiplication). Independent-samples *t* tests indicated that the mathematics achievement index was significantly greater in the math group than in the control group, *t*(69) = −5.81, *p*<.001, which was also the case for all math scores taken independently. On the other hand, both groups of participants presented similar scores at the WRAT4 Spelling test, *U* = 415.5, *z* = −1.6, *p*>.1 (see Table 1 for descriptive statistics). In the math group, undergraduate and postgraduate students presented similar mathematics achievement index, *U* = 94.5, *z* = −1.18, *p*>.2, and similar spelling scores, *t*(27) = −.70, *p*>.4. Preliminary analyses on all the following experiments and measures taken in this study indicated that undergraduate and postgraduate participants in the math group systematically presented similar results.

**Table 1:** Descriptive statistics of tests for mathematics achievement and spelling abilities by group of participants.

	Math	Group	Control	Group	Group	Difference
	Median (SD)	Min-Max	Median (SD)	Min-Max		
WRAT4-Spelling^a^	32 (3)	28–38	33 (2)	25–37	*U* = 415.50 *p*>.1	*z* = −1.58
WRAT4-Math^a^	34 (3)	25–38	27 (4)	18–35	*t*(69) = −6.10	*p*<.001
Addition^a^	28 (5)	15–44	24 (5)	15–34	*U* = 334.5 *p* = .001	*z* = −3.40
Subtraction^a^	25 (5)	10–41	21 (5)	15–33	*U* = 346.00 *p* = .001	*z* = −3.26
Multiplication^a^	32 (5)	25–43	23 (5)	14–35	*t*(69) = −6.55	*p*<.001
Calculation^a^	86 (13)	50–122	70 (13)	48–97	*U* = 253.50 *p*<.001	*z* = −4.31
Mathematics Achievement	30 (4)	20–40	25 (4)	17–33	*t*(69) = −5.81	*p*<.001

a Raw scores are reported here.

b Calculation corresponds to participants’ total score at the calculation test (i.e., addition, subtraction, multiplication).

c Mathematics Achievement Indices were computed by averaging the participants’ results at the WRAT4-Math test, the addition, the subtraction and the multiplication sections of the calculation test.

Correlational analyses across both groups of participants revealed significant positive correlations between the different mathematics achievement measures (all *p_s_*<.001) and the absence of significant correlations between the WRAT4 Spelling test and all the different math tests (all *p_s_*>.2).

These primary results are of great importance: they demonstrate that, despite both groups of participants being well educated in general and having had formal school education in mathematics, they showed significantly different levels of mathematics achievement in accordance to their different levels of experience in mathematics, while at the same time showing equal performance on a task tapping more general cognitive abilities.

## Experiment 1: Non-symbolic Comparison Task

### Method

#### Stimuli and procedure

This experiment is a replication of the larger-smaller behavioural task used in Piazza et al. [7], in which participants had to judge whether target magnitudes were smaller or larger than a reference number. As in Piazza et al. [7], two reference numbers were used: reference number 16 and reference number 32. On each trial a series consisting of four different sets of dots was presented, three with the same number of items (i.e., the reference number 16 or 32) and a fourth with a different number of items (12, 13, 14, 15, 17, 18, 19, 20 or 24, 26, 28, 30, 34, 36, 38, 40). Participants judged whether the last set had a larger or smaller number of items than the preceding ones. The experiment consisted of 8 blocks of 40 trials each, in which trials were randomly selected. There were four blocks with reference 16 and four with reference 32 randomly mixed together across participants.

The experiment was conducted on a PC computer. Each trial started with a fixation cross displayed for 1050 ms, each set of dots was then presented for 150 ms. Dots were white against a black background, followed by a fixation cross for 1050 ms before the next set of dots appeared. Stimuli were controlled for item size, inter-item spacing, total luminance and total occupied area as described in Piazza et al. [7]. The experiment started with 10 practice trials, which were not included in the analyses.

### Results

Following the data-trimming procedure, 2.01% of the data were taken out of the analyses, including reaction times (RTs) smaller than 150 ms as well as trials in which responses were outside 3 standard deviations around each participant’s average RT. The analyses were conducted on 66 participants: thirty-three were in the math group (1 participant missing: data not recorded) and thirty-three in the control group (4 participants excluded: accuracy below 70%). There was no speed accuracy trade-off in both groups of participants (*p_s_*>0.1).

First, we investigated the occurrence of a distance effect on RTs and accuracy. Second, correlation analyses were conducted to investigate whether participants’ level of mathematics achievement correlated with RTs, accuracy and the distance effect. Third, ANS acuity (*w*) was estimated and compared. Correlation analyses were then carried out to examine whether mathematics achievement correlated with ANS acuity.

#### The distance effect

**Figure 2:**
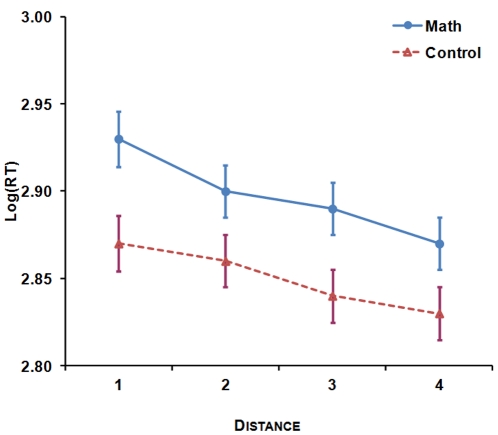
The distance effect on log(RT) by group in the non-symbolic comparison task.

To investigate the distance effect, two separate ANOVAs with Reference Number (2)×Numerical Distance (4)×Group (2) were conducted on log(RT). A logarithmic transformation was applied to RTs in order to meet the normality assumption in order to conduct analyses of variance. The results showed a significant effect of group, *F*(1,64) = 4.65, *p*<0.05, with participants in the control group presenting faster RTs on average than participants in the math group (control group: mean log(RT) = 2.85/RT = 725 ms; math group: mean log(RT) = 2.89/RT = 808 ms). Numerical Distance from the reference number also had a significant effect on log(RT), *F*(3,192) = 76.50, *p*<0.001, with longer RTs for sets of dots that were closer in numerosity to the reference number. Furthermore, the interaction between Numerical Distance and Group was significant, *F*(3,192) = 3.21, *p*<0.05 (see Figure 2). To further study this interaction, we computed for each reference number participants’ linear regression slope for RTs with distance as a predictor. These regressions’ slopes (*b*) can be taken as an index of the effect of distance on participants’ reaction times: the steeper the slopes, the larger the distance effect [47]. Because the regression slopes in the math group were not normally distributed, the non-parametric Mann-Whitney test was used to compare the slopes of control and math participants. The results indicated that the distance effect was more pronounced in the math group than in the control group for reference number 16, *U* = 377.00, *z* = −2.15, *p*<.05, *r* = −.26 (*Mdn* = −35.27 in the math group; *Mdn* = −20.37 in the control group); it tended to be more pronounced in the math group (*Mdn* = −17.89) than in the control group (*Mdn* = −13.82) for reference number 32, but not significantly, *U* = 428.00, *z* = −1.49, *p*>.1, *r* = −.18. The mean regression slopes across both reference numbers were significantly steeper in the math group (*Mdn* = −27.41), than in the control group (*Mdn* = −16.34), *U* = 384.00, *z* = −2.06, *p*<.05, *r* = −.25, confirming the occurrence of a greater distance effect on RTs in the math group compared to the control group.

Non-parametric analyses were conducted on accuracy scores, as the assumption of normality was violated. First, we conducted Mann-Whitney Tests for both reference numbers to study the occurrence of a group effect. The results showed that there was no group effect in terms of accuracy for both reference number, *U* = 538.5, *p*>.9 for reference number 16, *U* = 485.5, *p*>.4 for reference number 32. Second, the accuracy scores appeared to be slightly better for reference number 16 than for reference number 32, as indicated by the marginally significant Wilcoxon Tests in the math group, *z* = −1.33, *p* = .1 (Reference Number 16 *Mdn* = .84, *SD* = .05; Reference Number 32 *Mdn* = .83, *SD* = .06), and in the control group, *z* = −1.76, *p* = .07 (Reference Number 16 *Mdn* = .83, *SD* = .06; Reference Number 32 *Mdn* = .84, *SD* = .05). These results reflect the classic size effect: the smaller the target magnitude, the greater the accuracy [1]. Third, Friedman’s ANOVAs for both reference numbers with distance as test variable demonstrated a significant distance effect on accuracy for both reference numbers in both groups: for reference 16, χ^2^(3) = 83.32, *p*<.001 in the math group, χ^2^(3) = 74.04, *p*<.001 in the control group; for reference 32, χ^2^(3) = 78.43, *p*<.001 in the math group, χ^2^(3) = 88.13, *p*<.001 in the control group.

#### Mathematics achievement and non-symbolic comparison

Spearman’s correlational analyses across both groups of participants between mathematics achievement and the average non-symbolic comparison RTs, accuracy and distance slopes on RT were conducted for reference number 16 and 32. No correlation reached significance (*p*_s_>.1).

#### ANS acuity

As a measure of the precision of the underlying numerical representation we estimated the internal Weber fraction (*w*) for each participant following the procedure described in the Supplemental Data of Piazza et al. [7]. The median estimated Weber fraction (*w*) for reference number 16 was 0.14, for reference number 32 it was 0.15, resulting in an overall median estimate for *w* of 0.14. There was no group difference on the overall median estimate of the Weber fraction, *U* = 506.00, *z* = −.49, *p*>.6 (*Mdn w* = .140 in the math group, *Mdn w* = .139 in the control group).

#### ANS acuity and mathematics achievement

Spearman’s correlational analyses across both groups of participants between the mathematics index and overall *w* showed that mathematics achievement did not correlate with *w*, *r_s_* = −.13, *p*>.3. Correlational analyses were also conducted between the different mathematics achievement measures collected and overall *w*. All the correlations were non-significant (all *p*_s_>.1).

## Experiment 2: Symbolic Comparison Task

In experiment 1 we tested non-symbolic quantity comparison. In experiment 2 participants performed comparison on symbolic material (Arabic digits).

### Method

#### Participants

The math group in experiment 2 consisted of 34 students (23 males, 11 females; 2 left-handed) from the School of Mathematics at the University of Leeds, aged between 20 and 24 (*M* = 22, *SD* = 2). Half of these took part in all experiments. The control participants were 39 students (7 males, 32 females; 2 left-handed) in the Institute of Psychological Sciences at the University of Leeds aged between 20 and 29 (*M* = 22, *SD* = 2). Of these, 16 participated in all experiments. Participants were either undergraduate students in their final year or post-graduate students. New participants were tested on the WRAT4 arithmetic and spelling subtests, as well as on the calculation test. We replicated the previous results found with these two new groups of participants. Independent samples *t* tests showed that the mathematics achievement index was significantly greater in the math group (Math index = 29.47) than in the control group (Math index = 24.70), *t*(71) = −3.88, *p*<.001, while the WRAT4 Spelling scores were similar in both groups, *p*>.3. This was also the case, when only the new participants were included in the analyses: Math index = 29 in the math group vs. Math index = 24.5 in the control group, *t*(39) = −2.40, *p*<.05; WRAT4 Spelling score = 33 in both groups, *p*>.5.

#### Stimuli and procedure

The symbolic comparison task was a classic comparison task with a fixed reference number (65) [71]. In this task, all Arabic numerals from 31 to 99, except the standard 65, were presented to the participants. The 68 target numerals were presented 6 times, giving a total of 408 target stimuli divided into three experimental blocks. A pseudorandom list of the Arabic numbers was created so that each target number was presented twice in each block, and that the same number was never presented twice in a row. The experimental blocks were preceded by a training list of 10 numbers, which were not included in the analyses.

The experiment was conducted on a PC computer. Each trial started with a delay of 300 ms before the presentation of a fixation cross (500 ms), followed by a target number presented in Courier New 28 font for an unlimited duration. Participants had to press the right-hand key “l” on the computer keyboard if the number presented was larger than 65, and the left-hand key “a” if the number presented was smaller than 65. In the instructions, the experimenter emphasized both speed and accuracy.

### Results

0.5% of the data were taken out of the analyses, following the data-trimming procedure (i.e., a 3 standard deviation cut-off applied for RTs). There was no significant speed accuracy trade-off in the math group, *r = *.17, *p*>0.3. However, there was a significant speed accuracy trade-off in the control group, *r* = .42, *p*<.01.

#### The distance effect

First, the distance effect was investigated on RTs. In order to meet the normality assumption, a logarithmic transformation was applied to reaction times. The target numbers were clustered into four different distance bins: distance 1 included target numbers (61–64, 66–69) (i.e., 1 to 4 units distant from the standard 65); distance 2 included target numbers (51–60, 70–79) (i.e., 5 to 14 units distant from 65); distance 3 included target numbers (41–50, 80–89) (i.e., 15 to 24 units distant from 65); and distance 4 included target numbers (31–40, 90–99) (i.e., 25 to 34 units distant from 65). An ANOVA with Numerical Distance (4)×Group (2) was conducted on log(RT). Results showed a significant distance effect, *F*(3, 213) = 388.46, *p*<.001, with slower reaction times the smaller the distance between the target number and the standard 65. There was no significant interaction and no group effect (*p*_s_>.1).

**Figure 3:**
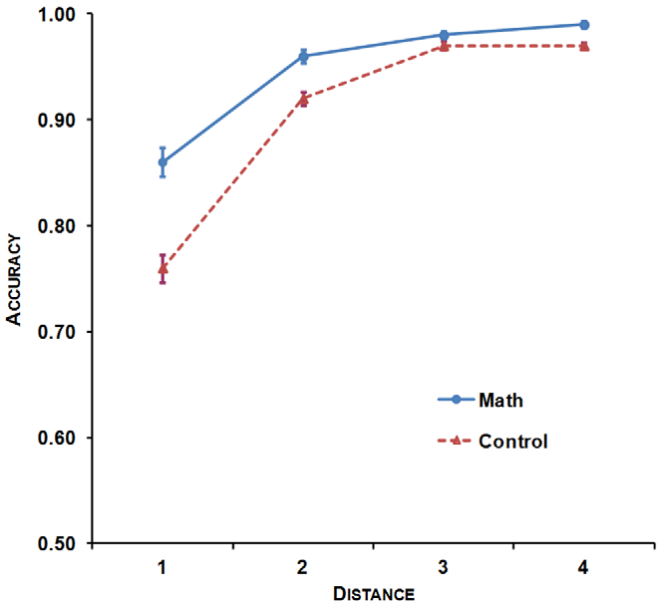
The distance effect on accuracy by group in the symbolic comparison task.

Second, the distance effect was measured on accuracy. Participants in the math group (*Mdn* = .96, *SD* = .03) presented significantly greater accuracy compared to participants in the control group (*Mdn* = .94, *SD* = .03), as indicated by the significant group effect, *U* = 264.5, *z* = −4.45, *p*<.001. Both groups of participants presented a significant distance effect on accuracy, χ^2^(3) = 75.03, *p*<.001 in the math group, χ^2^(3) = 103.61, *p*<.001 in the control group. To further study these primary results on the distance effect, participants’ linear regression slope for accuracy with distance as a predictor were computed as an index of the distance effect. Independent-samples *t* tests comparing the slopes of control and math participants showed that the symbolic distance effect was significantly more pronounced in the control group than in the math group, *t*(71) = 4.81, *p*<.001 (*b* = .005, *b* = .003 respectively) (see Figure 3).

#### Mathematics achievement and symbolic comparison

**Figure 4:**
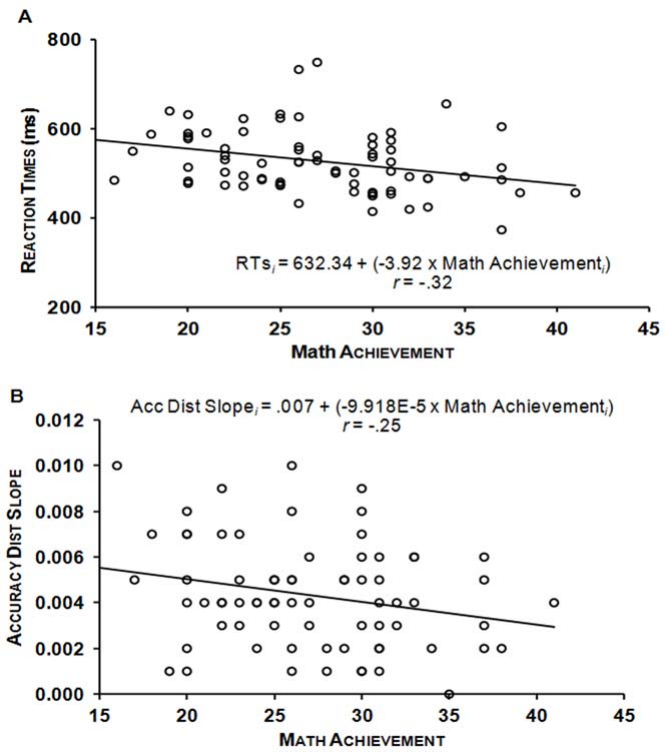
Scatterplots showing significant correlations between mathematics achievement and: (A) RTs; and (B) the distance slope for accuracy in the symbolic comparison task.

Mathematics achievement was significantly negatively correlated with RTs, *r* = −.32, *p*<.005 (all correlations were one-tailed, as positive relationship between mathematics achievement and symbolic numerical processing was expected [26], [43], [47], [48], [52]), as well as with the symbolic distance slopes, *r* = −.25, *p*<.05: the greater mathematics achievement, the faster RTs and the smaller the distance effect on accuracy (see Figure 4). As the number of males and females between both groups was not equally distributed and as gender differences have been found on intelligence [73], [74], mathematical reasoning [75] and symbolic magnitude estimation [76], we conducted hierarchical regression analyses. With these analyses, we investigated the extent to which participants’ mathematics achievement explains unique variance in symbolic comparison RTs and distance effect on accuracy, when controlling for possible effects of gender, but also of more general cognitive abilities that might be reflected in other cognitive skills such as spelling. We sequentially included three steps in the following hierarchical regression analyses: 1) gender; 2) WRAT4 Spelling score; 3) Mathematics achievement. These correlations remained significant for RTs, Δ*R^2^* = .09, Δ*F*(1, 63) = 7.33, *p*<.01, and marginally significant for the symbolic distance effect on accuracy, Δ*R^2^* = .04, Δ*F*(1, 63) = 3.22, *p* = .07, after controlling for gender and spelling. Secondly, accuracy in the symbolic comparison task correlated positively and significantly with mathematics achievement, *r_s_* = .31, *p*<.005: the greater mathematics achievement, the greater accuracy. This correlation remained significant after controlling for gender and spelling, Δ*R^2^* = .07, Δ*F*(1, 63) = 5.83, *p*<.05 (see Table 2).

**Table 2:** Hierarchical regression analyses to control for gender and general cognitive abilities.

		*Symbolic Comparison & Math Index*							
		*RTs*					*Accuracy*		
		*Beta*	*RSquare*		*RSquare* *Change*		*Beta*	*RSquare*	*RSquare Change*
*1.*	*Gender*	−.12	.00		.00		−.25	.10	.10*
*2.*	*Spelling*	−.30	.11		.11**		−.22	.14	.04
*3.*	*Mathematics achievement*	−.31	.21		.09**		.28	.21	.07*
			***Distance slope***						
			***Beta***	***RSquare***		***RSquare*** ***Change***			
*1.*	*Gender*		.21	.07		.07*			
*2.*	*Spelling*		.20	.10		.03			
*3.*	*w*		.22	.14		.04			
		***Numerosity Production and Math Index***							
		***Production [ER]*** ***Extensive Set***					***Production [ER]*** ***Intensive Set***		
		***Beta***	***RSquare***		***RSquare*** ***Change***		***Beta***	***RSquare***	***RSquare*** ***Change***
*1.*	*Gender*	−.18	.01		.01		.03	.01	.01
*2.*	*Spelling*	.04	.01		.00		.09	.01	.00
*3.*	*Mathematics achievement*	−.22	.06		.04		−.32	.11	.10*
		***Numerosity Production and Perception & Math Index***							
		***Perception [ER]*** ***Extensive Set***					***Perception [ER]*** ***Intensive Set***		
		***Beta***	***RSquare***		***RSquare*** ***Change***		***Beta***	***RSquare***	***RSquare*** ***Change***
*1.*	*Gender*	.16	.01		.01		.02	.00	.00
*2.*	*Spelling*	.08	.02		.01		.00	.00	.00
*3.*	*Mathematics achievement*	.21	.06		.04		.29	.08	.08*

* *p*<.05.

** *p*<.01.

*** *p*<.001.

## Experiment 3: Numerosity Production Task

In all previous experiments the stimulus material within each experiment was of the same type (e.g. groups of dots, Arabic digits). In the following experiments participants had to map between symbolic and non-symbolic number formats. Experiment 3 assessed their ability to produce non-symbolic quantities for given Arabic digits. We refer to this experiment as the production task from here on.

### Method

#### Participants

Participants were the same as in Experiment 1.

#### Stimuli and procedure

**Figure 5:**
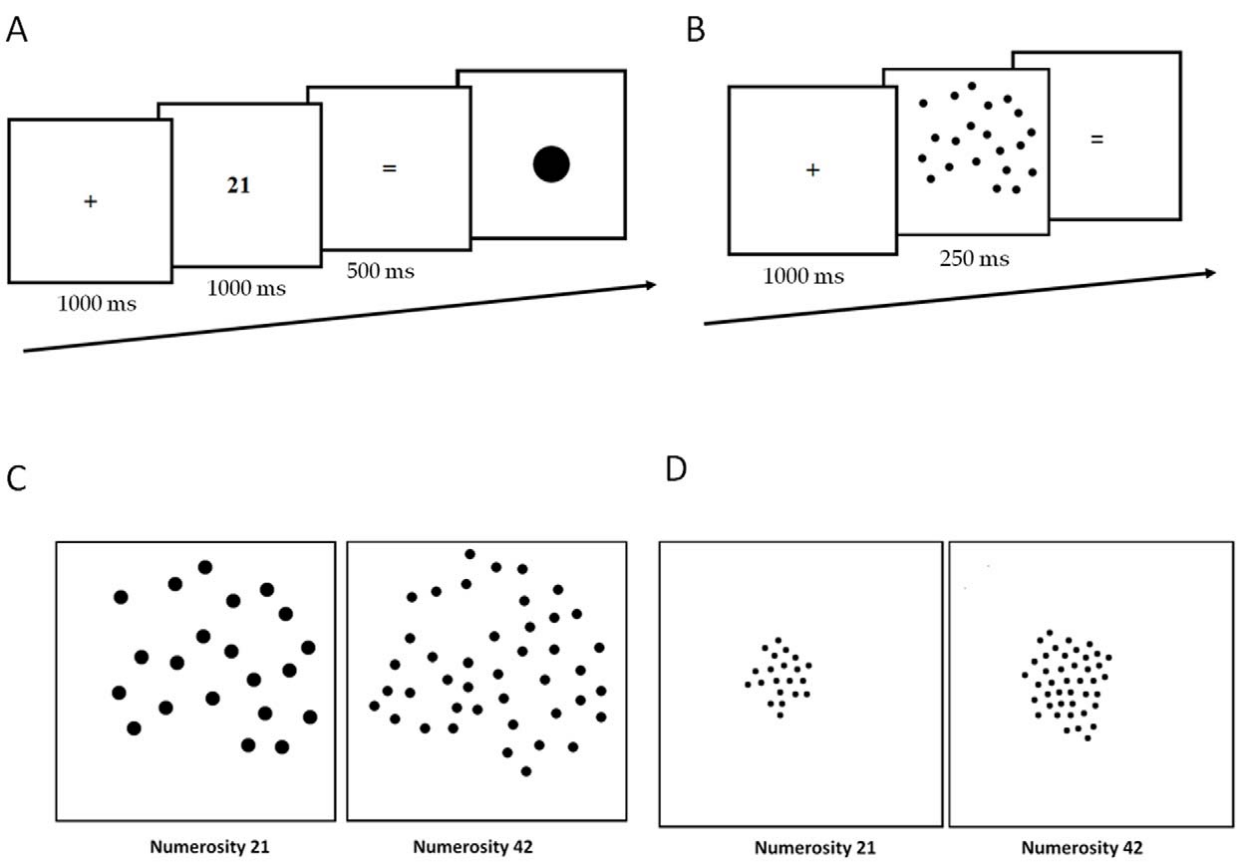
The numerosity production task (A) involving participants to perform a symbolic to non-symbolic mapping; and the numerosity perception task (B) involving participants to perform a non-symbolic to symbolic mapping. In the production task, the presentation of a single dot following the stimulus presentation corresponds to a signal for the participant to start their response production. Illustration of Extensive patterns of dots (C) and an Intensive pattern of dots (D) for numerosities 21 and 42.

The numerosity production task was an estimation task involving the production of non-symbolic quantities (i.e. sets of dots). Sixteen target numerosities were used: 21, 24, 27, 30, 33, 36, 39, 42, 49, 56, 63, 70, 77, 84, 91, 98. The experiment was conducted on a PC computer. Each trial started with a delay of 1000 ms before the presentation of a fixation cross (1000 ms), followed by a target Arabic number (1000 ms), followed by the symbol “ = ” (500 ms). Then a single dot appeared on the screen for an unlimited period of time (see Figure 5a). This single dot indicated participants to start their numerical estimation through the production of a set of dots of a quantity corresponding approximately to the target number presented. The same material as in Crollen et al. [18] was used: 1) participants had to produce their responses with the use of a potentiometer on a response box; 2) to control for low-level perceptual variables, half of the pattern of dots produced were initiated from an Extensive Set (i.e. luminance and total occupied area kept constant across numerosity) (see Figure 5c) and the other half from an Intensive Set (i.e. dots’ size and patterns’ density kept constant across numerosity) (see Figures 5d); 3) the number of dots that could be produced was limited to a maximum of 254 dots. The generated dots were black on a white background. The experiment was made of 8 blocks of 32 trials, preceded by 8 practice trials not included in the analyses. In each experimental block, the 16 target numerosities were randomly presented twice with production patterns initiated from both sets. The order of the blocks was counterbalanced across participants and across groups: half of the participants undertook block 1 to block 8, the other half undertook block 8 to block 1.

Participants were told that they would see Arabic numbers on the screen and that they should approximately produce their corresponding quantity with the use of a potentiometer on a response box. They had to report their completion by pressing a key on the response box. The use of a counting strategy was prevented, as dots were appearing very quickly on the screen while the participants were turning the potentiometer (one dot/1.4° of angular rotation). Moreover, although there was no time limitation for the response production, participants were required to perform the task under speed pressure and specifically instructed not to try using a counting strategy to solve the task. The experimenter emphasized the fact that the experiment was a numerical estimation task and that no accurate responses were expected. No information regarding the numerical range used was given.

### Results

The analyses on this experiment were conducted on 31 participants in the math group and 31 participants in the control group, as due to technical problems no responses were recorded for 9 participants (3 in the math group, 6 in the control group). Following the data-trimming procedure with a 3 standard deviation cut-off in each cell, 0.56% of the data were removed from the analyses. Preliminarily analyses indicated that stimuli from the Extensive and Intensive Sets appeared to present different levels of difficulty with greater error rates in the Intensive Set (*Mdn* = .82, *SD* = .42) than in the Extensive Set (*Mdn* = .66, *SD* = .40), *t*(61) = −6.48, *p*<.001. Therefore, the analyses were conducted separately for each set of stimuli.

First, coefficients of variation were computed and regression analyses were conducted to investigate whether participants’ mapping abilities presented the signature of Weber’s law [27]. Second, analyses of variance were carried out on the error rates to investigate the precision of participants’ mapping abilities. Third, the relationship between mathematics achievement and mapping performances was investigated with the use of correlation analyses.

#### Mapping abilities and Weber’s law

If participants’ performance obeys Weber’s law their mean responses and their standard variations should increased linearly and in direct proportion with target numerosity, so that coefficients of variation across target size (CV: ratio of the standard deviation and the mean response) should be constant. To determine whether participants’ patterns of performance obeyed Weber’s law, we conducted regression analyses on log(CV)s by stimulus set and by group. A logarithmic transformation of the CVs observed by participant was applied to equate variability in the estimated CVs [18], [27]. In the math group, participants presented constant log(CV)s as soon as the last target number was taken out of the analysis in the Extensive Set, *t*(15) = −1.01, *p*>.3, and as soon as the two last target numbers were taken out of the analysis in the Intensive Set, *t*(14 = −1.63, *p*>.1. In the control group, the log(CV)s got constant when the last target number was taken out in the Extensive Set, *t*(15) = −1.99, *p*>.05, but when the five last target numbers were taken out in the Intensive Set, *t*(10) = −2.21, *p*>.05. The response production limitation (i.e. maximum of 254 dots on the screen) probably accounted for the fact that the largest target number(s) needed to be taken out of the analyses in order for the coefficients of variation to be constant across target size. Indeed, the response production limitation constrained participants’ response variability for the largest target numbers. Independent-samples tests indicated that the math group (*Mdn* = −1.38, *SD* = .27 in the Extensive Set; *Mdn* = −1.37, *SD* = .22 in the Intensive Set) and the control group (*Mdn* = −1.45, *SD* = .18 in the Extensive Set; *Mdn* = −1.52, *SD* = .20 in the Intensive Set) showed similar log(CV) in both stimulus sets (*p_s_*>.2).

#### Mapping precision

Friedman’s ANOVAs per stimulus set were conducted in each group to measure the effect of target numbers on the error rate (ER = (response – target number)/target number). Mean error rates were computed by target number for each participant. Target numbers were then introduced in four target sizes: Num1 = (21, 24, 27, 30); Num2 = (33, 36, 39, 42); Num3 = (49, 56, 63, 70); Num 4 = (77, 84, 91, 98). The results indicated an effect of target number in both groups: in the Extensive Set, χ^2^(3) = 13.56, *p*<.005 in the math group, χ^2^(3) = 33.11, *p*<.001 in the control group; and in the Intensive Set, χ^2^(3) = 8.40, *p*<.05 in the math group, χ^2^(3) = 7.52, *p* = .05 in the control group. These effects of target number in both groups and both sets of stimuli reflect the classic observation, according to which the ER significantly increased with target number [18], [27]. This effect indicated that the larger the target number, the more participants over-estimated the number of dots to produce in order to reach its corresponding magnitude. In the two sets of stimuli, the math and the control participants presented similar ER (*p_s_*>.1): in the Extensive Set, Math *Mdn* = .66, *SD* = .38, Control *Mdn* = .73, *SD* = .41; in the Intensive Set, Math *Mdn* = .76, *SD* = .43, Control *Mdn* = .88, *SD* = .40.

#### Mapping abilities and mathematics achievement

**Figure 6:**
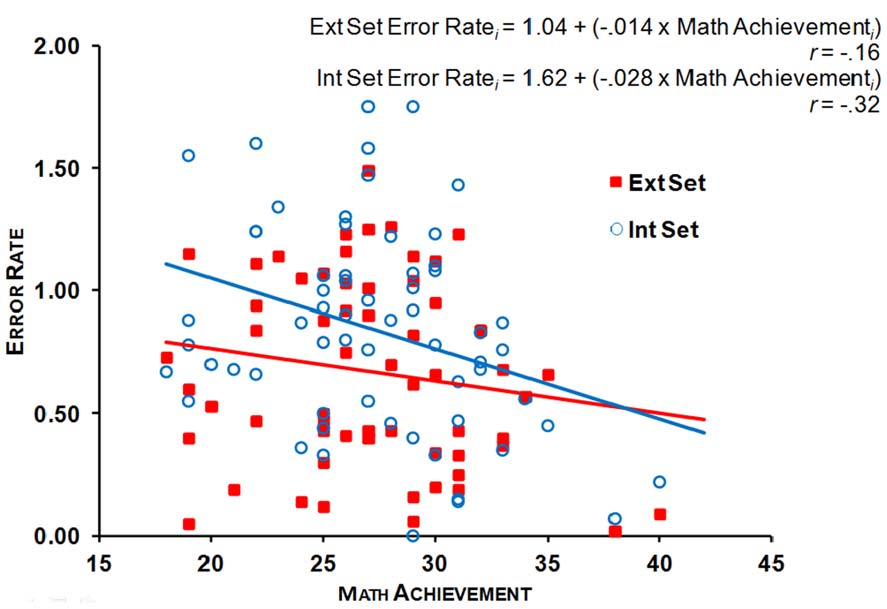
Scatterplot showing the correlations between mathematics achievement and ER (i.e. (response – target number)/target number) in the numerosity production task in the Extensive and Intensive Sets.

Correlational analyses per stimulus set across both groups of participants between mathematics achievement index and the numerosity production log(CV) and ER were conducted. These analyses showed that log(CV) did not correlate with mathematics achievement in both stimulus sets, *p_s_*>.2. On the other hand, the ER in the production estimation task significantly correlated with mathematics achievement in the Intensive Set: *r* = −.32, *p* = .01, and marginally correlated with mathematics achievement in the Extensive Set: *r* = −.16, *p* = .1: the better the level of mathematics achievement, the smaller the ER (see Figure 6). As previously, hierarchical regression analyses showed that mathematics achievement still accounted for a significant amount of variance in numerosity production ER when controlling for gender and spelling in the Intensive Set, Δ*R^2^* = .10, Δ*F*(1, 53) = 5.42, *p*<.05, and for marginal significant amount of variance in the Extensive Set, Δ*R^2^* = .04, Δ*F*(1, 53) = 2.38, *p* = .1 (see Table 2).

## Experiment 4: Numerosity Perception Task

In experiment 4, participants’ mapping abilities from non-symbolic numerosities (i.e., sets of dots) to symbolic numbers (i.e., Arabic digits) were assessed. We refer to this experiment as perception task from here on.

### Method

#### Participants

Participants were the same as in Experiments 1 and 3.

#### Stimuli and procedure

In the numerosity perception task participants had to estimate non-symbolic numerosities (i.e. sets of dots). The same sixteen numerosities as in the numerosity production task were used to generate the target sets of dots. The low-level continuous perceptual variables were similarly controlled by the introduction of two different sets of stimuli: extensive and intensive sets. A white background with black dots was used.

The experiment was conducted on a PC computer. A single trial started with a blank screen (1000 ms), followed by a central fixation cross (1000 ms). The stimulus display was presented for 250 ms, followed by the sign “ = ” that endured until a response was made (see Figure 4b). Participants responded by pressing the space key and simultaneously saying aloud their response (for a similar procedure, see [77]–[80] for examples). Participants were then prompted to encode their response on the number computer key pad. Eight practice trials were completed followed by 8 blocks of 32 test trials (2 stimulus sets×16 target numerosities). The stimuli were randomized within blocks. After each block, participants were given the opportunity to take a break. The order of the blocks was counterbalanced across participants.

### Results

The analyses on this experiment were conducted on 67 participants: 32 in the math group and 35 in the control group, as 2 control participants’ and 2 math participants’ responses were not recorded. Following the trimming procedure (i.e., a 3 standard deviation cut-off), 1.21% of the trials were not included in the analyses. Similar analyses as in the numerosity production task were conducted. As in the production task, preliminarily analyses indicated that stimuli from the Extensive and Intensive Sets appeared to present different levels of difficulty with greater error rates in the Intensive Set (*Mdn* = .37, *SD* = .16) than in the Extensive Set (*Mdn* = .35, *SD* = .14), *t*(66) = −2.55, *p* = .01. Therefore, the following analyses were conducted separately for each set of stimuli.

#### Mapping abilities and Weber’s law

The control group showed constant log(CV) when the last target numerosity (98) was taken out of the analysis in the Extensive Set, *t*(14) = −1.57, *p*>.1; and when the last two target numerosities (91, 98) were taken out of the analysis in the Intensive Set, *t*(13) = −1.12, *p*>.2. The math group showed constant log(CV) when all target numerosities were included in the analysis in the Extensive Set, *t*(15) = −1.23, *p*>.2; and when the last target numerosity (98) was taken out of the analysis in the Intensive Set, *t*(14) = −2.06, *p*>.1. No group difference on log(CV) was found between the math group (Extensive Set: *Mdn* = −1.57, *SD* = .21, Intensive Set: *Mdn = *−1.62, *SD* = .22) and the control group (Extensive Set: *Mdn* = −1.64, *SD* = .26, Intensive Set: *Mdn = *−1.59, *SD* = .24) in both stimulus sets (*p_s_*>.5).

#### Mapping precision

Friedman’s ANOVAs were conducted by stimulus set in each group to measure the effect of target numbers on the error rate (ER = (response – target number)/target number). As in the production task, mean ER were computed by target number for each participant and were then clustered into four target magnitude sizes. The results indicated an effect of target magnitude in both stimulus sets in the math group: χ^2^(3) = 30.69, *p*<.001 in the Extensive Set, χ^2^(3) = 53.08, *p*<.001 in the Intensive Set; as well as in the control group: χ^2^(3) = 37.40, *p*<.001 in the Extensive Set, χ^2^(3) = 70.62, *p*<.001. These results reflect the fact that ER significantly increased with target number in both groups: the larger the target number, the more participants under-estimated the number of dots presented [18], [27]. Independent-samples tests indicated the control group presented greater ER than the math group. Indeed, a significant group effect was found in the Intensive Set, *t*(65) = −2.52, *p*<.05 (*Mdn* = −.30, *SD* = .16 in the math group; *Mdn* = −.38, *SD* = .14 in the control group); and a marginally significant group effect was found in the Extensive Set, *U* = 457.50, *z* = −1.29, *p* = .1 (*Mdn* = −.30, *SD* = .13 in the math group; *Mdn* = −.40, *SD* = .15 in the control group).

#### Mapping abilities and mathematics achievement

**Figure 7:**
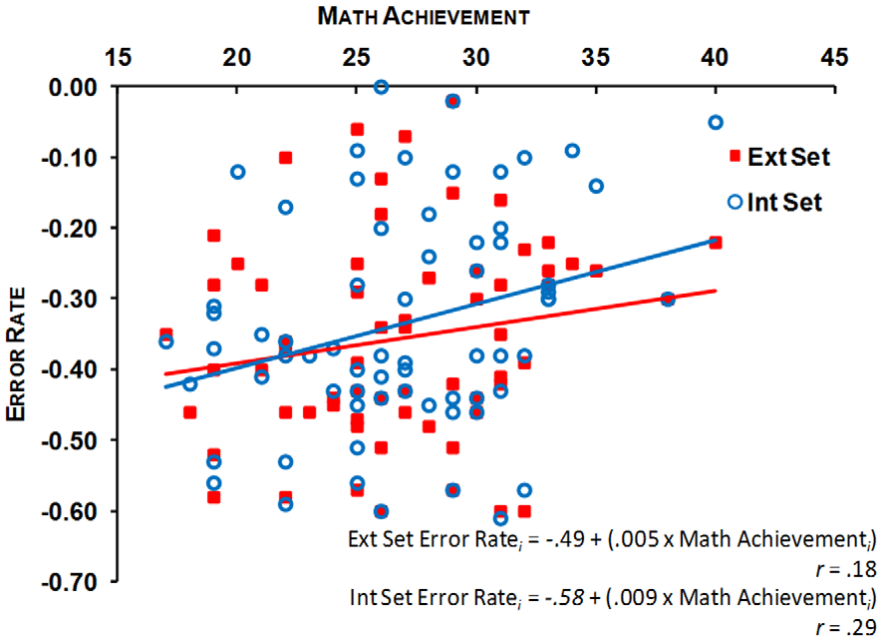
Scatterplot showing the correlations between mathematics achievement and ER in the numerosity perception task in the Extensive and Intensive Sets.

Correlational analyses per stimulus set across both groups of participants between mathematics achievement index and the numerosity production log(CV) and ER were conducted. These analyses showed that log(CV) did not correlate with mathematics achievement in both stimulus sets, *p_s_*>.2. On the other hand, the ER in the perception estimation task significantly correlated with mathematics achievement in the Intensive Set: *r* = .29, *p*<.01, and marginally correlated with mathematics achievement in the Extensive Set: *r* = .18, *p* = .07: the better the level of mathematics achievement, the smaller the ER (see Figure 7). Hierarchical regression analyses showed that mathematics achievement still accounted for a significant amount of variance in numerosity perception ER when controlling for gender and spelling in the Intensive Set, Δ*R^2^* = .08, Δ*F*(1, 58) = 4.96, *p*<.05, and for marginal significant amount of variance in the Extensive Set, Δ*R^2^* = .04, Δ*F*(1, 58) = 2.62, *p* = .1 (see Table 2).

### Correlational Analyses

As the same groups of participants took part in the different numerical tasks, one-tailed correlation analyses were conducted to investigate whether participants’ performances were correlated in the different tasks.

One-tailed correlational analyses between the numerosity perception and production tasks indicated that participants’ performances were significantly correlated in both numerical estimation tasks on non-symbolic material in both stimulus sets: the greater the variability (i.e. log(CV)) in numerosity production experiment, the greater the variability in the numerosity perception experiment, *r_s_* = .27, *p*<.05 in the Extensive Set, *r* = .32, *p*<.01 in the Intensive Set; the larger the [ER] (i.e., absolute value of ER) in the numerosity production experiment, the larger the [ER] in the numerosity perception experiment, *r_s_* = .30, *p* = .01 in the Extensive Set, *r* = .30, *p*<.05 in the Intensive Set.

Performances on symbolic and non-symbolic material were also significantly correlated. First, performance in the symbolic comparison task and the non-symbolic comparison task was correlated: 1) the faster the RTs in the non-symbolic comparison, the faster the RTs in the symbolic comparison task, *r_s_* = .78, *p*<.001, and the smaller the accuracy in the symbolic comparison, *r* = .33, *p*<.05; 2) the greater the accuracy in the non-symbolic comparison, the faster the RTs in the symbolic comparison, *r_s_* = −.29, *p* = .05. The symbolic and non-symbolic distance effects were correlated: the greater the distance effect on accuracy (i.e., regression slope) in the symbolic comparison, the smaller the distance effect on RT in the non-symbolic comparison task, *r_s_* = .33, *p*<.05. This correlation is due to the fact that, in the non-symbolic comparison task, the non-symbolic distance effect on RTs was more pronounced in the math group; while, in the symbolic comparison task, the symbolic distance effect on accuracy was more pronounced in the control group. This particular pattern of performance is further discussed in the following section.

A set of one-tailed correlational analyses between overall *w* and performances in the different numerical tasks on symbolic and non-symbolic material showed that the smaller participants’ *w*: 1) the faster the RTs in the symbolic comparison task, *r_s_* = .33, *p*<.05; 2) the smaller the variability (i.e., log(CV)) in the numerosity production task (Extensive Set, *r_s_* = .30, *p*<.05 and Intensive Set, *r_s_* = .30, *p* = .01); as well as in the perception task (Extensive Set, *r_s_* = .41, *p*<.001 and Intensive Set, *r_s_* = .35, *p*<.005); 3) the smaller the ER in the production task for the Intensive Set (*r_s_* = .27, *p*<.05), and in the perception task (Extensive Set, *r_s_* = .17, *p* = .09, and Intensive Set, *r_s_* = .18, *p* = .08, marginal significance).

## Discussion

Recently the relationship between basic number processing and mathematical skill has received a lot of attention [2], [26], [47], [48]. This study is to our knowledge the first to investigate the effects of an extended education in mathematics on the two core number systems, the ANS and the ENS, in adults. Our study shows that an extended education in mathematics appears not to be reflected in the acuity of the non-symbolic number system (i.e., the ANS). It seems, however, to be associated with a better anchored symbolic number system (i.e., the ENS) and more precise mapping abilities between non-symbolic and symbolic quantities. There are four main findings from the experiments presented above. First, ANS acuity is not linked with the level of of mathematics achievement in adults, because first the mean ***ANS acuity*** was similar for both groups of adults (around 0.15), whether they were undergoing further math education or not, and second ANS acuity was not significantly correlated with mathematics achievement. Moreover, our results replicated the average *w* of 0.15 repeatedly found for educated numerate adults [2], [6], [26]. Secondly, ***mathematics achievement*** ** ***and symbolic numerical abilities*** were significantly correlated. Thirdly, on the link between ***mathematics achievement and the distance effect***, the direction of the correlation appears to differ, whether the distance effect is on non-symbolic or symbolic numerical material. Last, ***mathematics achievement and mapping abilities*** are correlated. These findings will be discussed in turn.

### ANS Acuity

According to our results, individual differences in the quantity and quality of engagement in formal mathematics might not necessarily be associated with better ANS acuity per se, as it has been proposed in the literature [2], [8]. This important result does not simply correspond to the acceptance of a null hypothesis. On the contrary, it replicates and extends previous findings in developmental numerical cognition. Indeed, as in studies by Piazza et al. [26] and Mazzocco et al. [52] for children without mathematical learning difficulties, we failed to find, here in adults, clear evidence of a correlation between the ANS acuity (*w* in the non-symbolic comparison task; log(CV) in the numerosity production and perception tasks) and mathematics achievement. At this stage, it is important to emphasise that our results are not contrary to Halberda et al.’s [8] results. Indeed, Halberda and colleagues essentially found a correlation between 14 year olds’ ANS acuity and their *past* scores in math. Likewise, Mazzocco et al. [53] found a retrospective correlation between ANS acuity and mathematics achievement. Therefore, our results, together with Halberda et al.’s [8] and Mazzocco et al.’s [53] results, suggest that at some point in development, individuals seem to have reached their maximal ANS acuity with the ability to discriminate between sets with a 7∶8 ratio. Our results further suggest that individuals reach this maximal ANS acuity whatever their level of mathematics achievement.

Our results confirm the pervasiveness of an estimated *w* around 0.15 in adults [2], [7], [11], [26] independent of their level of mathematics achievement. These results are important as they demonstrate for the first time in adults, that the degree of involvement in adult math education seems to have no impact on the non-symbolic logarithmic core sense of approximate numerosity. As a consequence, they are in agreement with the suggestion of a universal *w* with a mean around 0.15 in adults [5], [11], [26]. However, our data does not exclude that, at earlier stages of mathematical development, differences in ANS acuity could be found. For example, it is possible that adults with higher mathematics achievement might reach their maximal ANS acuity earlier in development or might show higher ANS acuity when they start schooling compared to adults with lower mathematics achievement. This hypothesis appears to be highly possible, considering Mazzocco et al.’s [53] results showing that mathematics ability for children in primary school can be predicted by their ANS acuity at preschool. However, despite undergoing intensive training in mathematics and scoring significantly higher in mathematics achievement tests, adults in the math group showed essentially the same ANS acuity as adults in the control group, while clearly demonstrating better performances in basic numerical skills on symbolic numerals, as well as better mapping abilities between symbolic numerals and their corresponding magnitudes.

### Mathematics Achievement and Symbolic Number Processing

Although there was no link between ANS acuity and mathematics achievement, our data demonstrated clearly that an education focused on mathematics is reflected in basic numerical skills on symbolic material. Our findings are in line with previous findings in children (see [43], [47], [48], and see [26], [50], [52] for studies on learning disability). Mathematics achievement in adults was significantly correlated with basic numerical skills on symbolic numbers: the greater the mathematics achievement, the faster the RTs, the greater the accuracy and the smaller the distance effect on accuracy in the symbolic comparison task. Therefore, our results give further support to the idea that the acquisition of the symbolic number knowledge leads to lasting changes in numerical processing, with the refinement of the logarithmic ANS into a linear exact number system (ENS) [6], [21]–[22], [33], [54], [59]. Indeed, the fact that participants with an education dedicated to mathematics showed better performance on a basic symbolic comparison task suggests that their greater experience with the symbolic number system and arithmetic has probably given rise to a better anchored ENS, where magnitudes can be directly accessed from symbolic numbers.

In addition, our findings support previous observations made in children, according to which the positive impact of greater mathematics achievement seems not to generalize to numerical processing on non-symbolic material [43], [48], [49]. Indeed, our results showed that mathematics achievement did not correlate with performances in the non-symbolic comparison task, except for the distance effect.

### Mathematics Achievement and the Distance Effect

Our study also showed a relationship between the distance effect and mathematics achievement. Contrary to some previous findings [45], [47], [48], our results demonstrate that the link between the distance effect and mathematics achievement is not only found at the early stages of formal mathematics instruction [44], [47], [48] and it appears not to be limited to symbolic material [43], [48]. However, the relationship between the distance effect and mathematics achievement seems to be complex and its direction to depend on the type of numerical material involved (symbolic vs. non-symbolic). Indeed, on the one hand, when processing non-symbolic numerical information, mathematics achievement was positively correlated with the distance effect on reaction times. On the other hand, when processing symbolic numerical information, mathematics achievement was negatively correlated with the distance effect on accuracy. We are now discussing these findings in view of the recent findings in the literature, separately for the symbolic and non-symbolic comparison tasks, in order to interpret this particular pattern of performances.

#### Mathematics achievement and the symbolic distance effect

In the symbolic comparison task, a classic distance effect was found on RTs and on accuracy in both groups of participants: the smaller the numerical distance between the numbers to process, the slower and the less accurate were the responses. The distance effect in response times did not differ between the groups. But interestingly, the control group showed a stronger distance effect for accuracy than the math group. The fact that both groups of participants presented similar RTs suggests that the difference found on the symbolic distance effect between the two groups did not stem from the use of different response strategies, but rather reflects differences in the underlying internal representation used. Control participants made more mistakes in symbolic comparison and showed a stronger distance effect for accuracy. This suggests that participants in the math group might have a better anchored representation for symbolic numbers accounting for more accurate performance and might be more able to adapt their ENS to the task at hand than participants from the control group. This result again reinforces the idea of an ENS that can be directly accessed from symbolic input [43], [79] and becomes better defined with increasing mathematics knowledge.

Therefore, although the linkage between the symbolic distance effect and mathematics achievement has been reported from childhood up into adulthood, the locus of this link might differ with age [47], [48]. In children, the association between the symbolic distance effect and mathematics achievement is particularly dominant in response times: better mathematics achievement in children is associated with a greater fluency/speed in accessing magnitude information from symbolic numerical representations. As a consequence, it has been postulated that early formal mathematics instruction leads to better mapping abilities between symbolic numbers and their corresponding magnitudes [47], [48]. In adults, according to our results, the association between mathematics achievement and the symbolic distance effect is noticeable on accuracy levels, suggesting the use of a better anchored underlying representation (i.e., the ENS). This result gives further evidence to the developmental proposal according to which, with age and increasing mathematics knowledge, better mapping abilities between the symbolic and non-symbolic numerical representations give rise to a better build-up ENS, directly accessed from symbolic input [43], [79].

#### Mathematics achievement and the non-symbolic distance effect

In the non-symbolic comparison task, both groups showed significant distance effects for response times and accuracy levels. However, this time their distance effects for accuracy levels were not significantly different. But there was a significant difference between the groups for the distance effect in response times, with the math group presenting a stronger distance effect on RTs. Moreover, the math group presented slower RTs than the control group.

Overall, a possible explanation to the observed difference in response times between both groups could be that participants from the math group might have used a different strategy (e.g., introduction of a non-symbolic to symbolic transcoding stage) than participants in the control group to solve the task, perhaps following a greater propensity to initiate automatic transcoding when encountering non-symbolic magnitudes. This hypothesis is supported by Gilmore et al.’s [19] results showing that higher achievers in mathematics showed a greater ratio effect in a non-symbolic addition task. The ratio effect, considered as a signature of Weber’s law, reflects the numerical distance and the size effect. It corresponds to a decrease of accuracy as the ratio between the quantities to compare approaches 1 [8]. For example, in Gilmore et al. [19], children had more difficult to compare numerosities differing by a ratio of 4∶5, compared to numerosities differing by a ratio of 4∶7. Following their results, Gilmore et al. [19] predicted as Halberda et al. [2] that children with greater mathematics achievement would present greater ‘sensitivity’ to non-symbolic numerical magnitudes. According to these authors, greater ‘sensitivity’ in higher achievers may involve greater motivation to attend to symbolic numerical representations in non-symbolic numerical tasks (i.e., transcoding activity). Therefore, in our study, the reaction time differences between the groups observed in the non-symbolic comparison task could be interpreted as resulting from extra processing time for converting non-symbolic into symbolic numerosities in the math group, which could in turn explain the greater distance effect on RTs found in this particular group. Indeed, the stronger distance effect on RT in the math group could be explained by an additional processing time used to transcode the non-symbolic numerosities into their corresponding symbolic representations. The stronger distance effect in RT then would be an additive effect of the distance effect for symbolic and non-symbolic numerosities which might be due to the sequential use of ANS and ENS. However, in view of the current literature and our results, further research would be needed to confirm this possible explanation.

In summary, higher mathematics achievement appears to lead to a stronger distance effect on response times for non-symbolic number comparison and a weaker distance effect on accuracy levels in symbolic number comparison. At first sight these findings seem contradictory, but they replicate behavioural results in children on symbolic material, where mathematics achievement negatively correlated with the symbolic distance effect [43], [48] and on non-symbolic material where mathematics achievement has been found to positively correlated with the ratio effect [19]; and they are in accordance with neuroimaging results, as adults present a greater non-symbolic distance effect at a neuronal level than children [81].

Our results strengthen the hypothesis that, in addition to presenting a more precise ENS, individuals with greater mathematics achievement might also possess a greater potential to automatically activate transcoding when encountering non-symbolic magnitudes [2], [19], leading to the use of different strategies for non-symbolic numerical processing, which might involve a wider, possibly more automatic recruitment of the ENS (e.g., introduction of a non-symbolic to symbolic transcoding stage). Additional support for this hypothesis is provided by the association found between mathematics achievement and mapping abilities between symbolic and non-symbolic quantities.

### Mathematics Achievement and Mapping between Symbolic and Non-symbolic Quantities

Mathematics achievement was correlated significantly with participants’ mapping abilities from symbolic to non-symbolic numerosities (numerosity production task) and from non-symbolic to symbolic numerosities (numerosity perception task): the greater the mathematics achievement, the smaller the over-estimation in the numerosity production and the smaller the under-estimation in the perception tasks. The ability to map between the symbolic and non-symbolic numerical representations develops between 6 and 8 years of age and is positively related to children’s mathematics achievement [13], [24], [43], [47], [48], [52]. Recently, Mazzocco et al. [52] further demonstrate that greater mathematics achievement in 14–15 year-olds is associated with a higher precision in their mapping abilities from non-symbolic to symbolic numerical presentations. Our results extend these findings to adults: the greater the mastery of the symbolic number system following an education dedicated to mathematics, the better the mapping abilities between symbolic numerical representations and their corresponding magnitudes. However, currently the question about the direction of this relationship and whether this relationship is causal is still open. Indeed, none of the existing studies can answer whether better mapping abilities are a consequence or a precursor of a more intensive use of the symbolic number system [52]. It is possible that stronger links between non-symbolic and symbolic number representations lead to a better symbolic number system. Alternatively, a well-developed symbolic number system might lead to a stronger link between the non-symbolic and symbolic number representations.

A modest impact of low-level continuous perceptive variables on numerical performance has been found in the production and perception tasks, with stimuli from the Intensive Set leading to greater error rates than the stimuli from the Extensive Set. The methodology of equating on half of the stimuli the extrinsic variables but randomly varying the intrinsic variables, while doing the reverse on the other half of the stimuli has been repeatedly used (e.g., [3], [7], [21], [82]), but still, as in our study, perceptual variables can impact the numerical processing in place (e.g., [7]). The influence of perceptual variables on non-symbolic numerical processing is a recurrent problem in the literature on numerical cognition (see [83]). Indeed, a facilitation effect when participants can rely on perceptual features, such as dot size (e.g., in the Extensive Set, dot size increases with increasing numerosity), has already been found in the literature (e.g., [24], [43]). Nevertheless, despite this perceptual bias, the observation of the signature of Weber’s law (i.e., constant CV) and the classic size effect (i.e., larger ER with larger magnitude) [18], [27], added to the fact that these results were consistent across stimulus sets and tasks, strongly suggest that participants were actually estimating numerical quantity rather than using perceptual variables in the production and perception task.

The consistency of our results within the current study and compared to other studies attests for their relevance and strength. Indeed, we firstly replicated classical numerical effects, such as the symbolic distance effect, as well as the non-symbolic distance and size effects [84]; the signature of Weber’s law for numerical processing: constant coefficient of variation across target size in the numerosity production and perception tasks [9] and a Weber fraction of 0.15 in the non-symbolic comparison task [2], [7], [11], [26]; and the classical opposite pattern of performance found according to the direction of the mapping involved in a numerical task, as suggested by the bi-directional mapping hypothesis: over-estimation when mapping from symbolic to non-symbolic vs. under-estimation when mapping from non-symbolic to symbolic [18], [27]. Moreover, within our study, participants present consistent results with significant correlations found between performances in the symbolic and in the non-symbolic comparison tasks, the different measures of ANS acuity (w in the non-symbolic comparison task and Log(CV) in the numerosity production and perception tasks); as well as performances in the numerosity production and perception tasks.

In summary, our results support the idea that two numerical systems might be at work in adults’ number processing: the ANS and the ENS (e.g., [6]). There is no doubt that the ANS acuity increases with age [5], [26] with a likely maximum acuity reached before or during early adulthood. Our study has shown clearly that the ANS in adults is independent of math achievement and experience with symbolic number representations. Our results thus support the hypothesis that ANS development might mainly be driven by a maturational process [5], [26], whatever individuals’ culture and/or level of math education. The acquisition of the symbolic number system and arithmetic is associated with better mapping abilities between the symbolic numerical representations and their corresponding magnitudes [13], [24], [43] and accompanied by a refinement of the logarithmic sense of approximate numerosity into a linear ENS, possibly located in the left IPS [5]–[6], [33], [59]. In contrast to the ANS, the ENS is amenable to education and cultural influences and is significantly related to mathematics achievement: the greater mathematics achievement, the stronger the mapping abilities and the better anchored the ENS is. In adults, ANS and ENS are two linked but separate systems. This does however not exclude that there might be a period during numerical development in which the ENS is strongly dependent on a well-developed ANS. Developmental studies are needed to shed further light on the relationship between ANS and the ENS, in particular focusing on the time interval when the ENS first emerges in children.
